# Overlap between adverse events (AEs) and serious adverse events (SAEs): a case study of a phase III cancer clinical trial

**DOI:** 10.1186/s13063-020-04718-z

**Published:** 2020-09-17

**Authors:** Elizabeth C. James, David Dunn, Adrian D. Cook, Andrew R. Clamp, Matthew R. Sydes

**Affiliations:** 1 MRC Clinical Trials Unit at UCL, Institute of Clinical Trials & Methodology, UCL, London, UK; 2 The Christie NHS Foundation Trust and University of Manchester, Manchester, UK

**Keywords:** Adverse events, Serious adverse events, Safety data

## Abstract

**Background:**

Safety data is required to be collected in all clinical trials and can be separated into two types of data, adverse events and serious adverse events. Often, these types of safety data are collected as two discrete data sets, where adverse events that also meet the criteria for seriousness should be reported in both datasets. Safety analyses are often conducted using only the adverse event dataset, which should feature all safety events reported. We investigated whether the reporting of safety in both datasets was systematically followed and explored the impact of this on safety analyses in ICON8, an ovarian cancer clinical trial.

**Methods:**

Text searches of serious adverse event data identified events that could potentially match the data reported in the adverse event dataset (looking at pre-specified AE terms only). These serious adverse events were then mapped to adverse event data according to predefined criteria: (a) event term matches, (b) date of onset and date of assessment within 30 days of each other, (c) date of assessment lies between date of onset and date of resolution and (d) events confirmed to occur in the same chemotherapy cycle. A combined dataset of all unique safety events (whether originally reported in the adverse event or serious adverse event dataset) was created and safety analyses re-performed.

**Results:**

51,019 adverse events were reported in ICON8, of which 42,410 were included in the mapping exercise. One thousand five hundred six serious adverse event elements were reported, of which 668 were included in the mapping exercise. Sixty-one percent of serious adverse event elements was matched to an already-reported adverse event. Supplementing these additional safety events and re-performing safety analyses increased the proportion of patients with at least one grade 3 or worse safety events in all arms from 42 to 47% in the control arm and 61 to 65% and 52 to 59% in the research arms. The difference in proportions of grade 3 or worse event in the research arms compared to the control arm changed by 18% (95% confidence interval [CI] 12 to 24%) and 12% (95% CI 6 to 18%), respectively.

**Conclusions:**

There was low agreement in mapping serious adverse events to already reported adverse events, with nearly 40% of serious adverse events included in the mapping exercise not mapped to an already reported adverse event. Any analyses of safety data that use only adverse event datasets or do not clearly account for serious adverse event data will likely be missing important safety information. Reporting standards should make clear which datasets were used for analyses.

## Introduction

Safety data sometimes referred to as ‘toxicity data’ or ‘side-effects’ can generally be separated into two types of events—adverse events (AEs) and serious adverse events (SAEs). These are defined in Table 1. SAEs must be collected as part of the regulatory requirements for a Clinical Trial of an Investigative Medicinal Product (CTIMP) [1]. If an event is deemed to meet the ‘seriousness’ criteria, further assessment is needed to determine causality and expectedness. Causality splits SAEs into unrelated SAEs (uSAE, not related to trial treatment) or a serious adverse reaction (SAR, related to trial treatment). Unexpected splits out a subset of SARs as suspected unexpected serious adverse reactions (SUSARs—related to trial treatment but not expected according to the contemporary reference safety information).

**Table 1:** Definition of an adverse event and a serious adverse event

Term	Definition
Adverse event (AE)	Any untoward medical occurrence in a patient or clinical investigation subject administered a pharmaceutical product and which does not necessarily have to have a causal relationship with this treatment.
Serious adverse event (SAE)	Any untoward medical occurrence that at any dose: a) Results in death b) Is life threatening c) Requires inpatient hospitalisation or prolongation of existing hospitalisation d) Results in persistent or significant disability/incapacity e) Is a congenital anomaly/birth defect (SAEs will be a subset of AEs)

Definitions as described by the International Council for Harmonisation (ICH)

Whether adverse events (AEs) have occurred is commonly solicited during routine assessment according to a predefined list of potential AEs of particular interest to a clinical trial [2]. These are assessed, categorised and graded at predefined time points across all arms of the trial against set criteria, such as the Common Terminology Criteria for Adverse Events (CTCAE) [3] or the Medical Dictionary for Regulatory Activities (MedDra). In contrast, SAEs are collected as and when they occur throughout the trial’s reporting period. After a pre-defined time point, only SARs and SUSARs continue to require reporting. SAEs are not be collected from a predefined list of events, and are usually reported in a free-text fashion, which may then be systematically coded at a later date.

In the cancer clinical trials at the Medical Research Council Clinical Trials Unit at UCL (MRC CTU), AEs and SAEs are usually collected on separate case report forms. Causality and expectedness are only collected for SAEs, not AEs, in order to reduce the data burden on sites. This means that any event that is on the predefined list of adverse events of particular interest and is ‘serious’ should be reported on two separate forms: once within 24 h of the site becoming aware the event has occurred as an SAE and once on the routinely collected AE section of the next assessment. Therefore, there will be an element of duplication in the safety events that are collected in such a trial. Theoretically, analyses that look at only the AEs dataset should give a true representation of all the safety events that were of particular interest before the trial, i.e. all SAEs should also already appear in the AE dataset. However, we hypothesised that not all such safety events would be correctly reported as both AEs and SAEs. If this hypothesis were correct, to look at one source of safety event would result in under-reporting the relevant safety events. Here, we explore the extent of this under- or over-lap of safety datasets in an ovarian cancer clinical trial and how this could influence the previously reported safety analyses.

## Methods

### ICON8

The International Collaboration on Ovarian Neoplasm 8 (ICON8) trial (ISRCTN10356387) was a phase III, three-arm randomised controlled trial for patients with epithelial ovarian, fallopian tube or primary peritoneal cancer [4]. The trial compared three chemotherapy regimens using 6 cycles of carboplatin and paclitaxel given on different fractionation schedules. These are widely used drugs with well-known toxicity profiles and many AEs and SAEs were expected. In the standard treatment, carboplatin and paclitaxel were both given every 3 weeks. In the first research treatment, carboplatin was given every 3 weeks and paclitaxel every week. In the second research treatment, both drugs were given every week. One thousand five hundred sixty-six participants were recruited from 2011 to 2014. Further details of the trial design and progression-free survival results have been reported previously [4]. The trial protocol is available on the trials unit website (https://www.ctu.mrc.ac.uk/studies/all-studies/i/icon8/). All data were collected on paper case report forms (CRFs).

### Adverse events

AEs were collected on the chemotherapy CRF according to CTCAE version 4.0. Events were graded for severity from 0 to 5 using CTCAE criteria, with 0 meaning the event has not occurred and 5 meaning the patient has died [3]. An assessment was performed by the site team on day one of the cycle of chemotherapy, and the worst grade of each event category since the last assessment was collected. The only date recorded is the assessment date; the exact onset and resolution dates of individual AEs were not collected. A list of 31 specific AEs was assessed at each assessment, and there was also space for ‘other’ events to be recorded, which were done so in free-text format. AEs were not solicited during post-chemotherapy follow-up. The CRF was often completed retrospectively, using patient notes as source data.

### Serious adverse events

SAEs were collected in ICON8 using the Unit’s standard SAE CRF, created in line with the Council for International Organizations of Medical Sciences (CIOMS) form [5]. This form collects detailed data on each serious safety event, including basic patient details, the reason for seriousness, where the SAE took place and details of the main symptom and any other associated events that occurred alongside. These details include CTCAE grade, dates of onset and resolution and event status (ongoing/resolved/resolved with sequelae) and, importantly, an assessment of causality and expectedness. A free-text narrative of the event is also recorded to give further information on the manifestation of the event, any treatments given in response and any tests performed. An SAE form may be sent to the MRC CTU partially completed when the site first become aware of the event, and then further updates sent as the event evolves over time. Event names are recorded in free-text format on the SAE form, which need to be categorised. SAEs were required to be reported to the CTU from the date the patient consented to join the trial, until 30 days post the last administration of trial chemotherapy. There is currently no inbuilt system to aid the coding of events in ICON8.

### Comparison of SAEs and AEs

A post hoc analysis was performed to compare the SAEs and AEs reported in ICON8. Text searches of all SAEs event terms were automated to identify if they matched any of the AE event terms already recorded in the routinely solicited assessments. Both main and associated events of each SAE were included in this analysis. The programme made allowances for spelling mistakes, alternative spellings for the same term and alternative medical terms; full list of free-text searches is in Supplementary Table 1. All categorisations were manually checked for misclassification and hard coding was performed where necessary. SAEs could match more than one AE if the event was ongoing over a number of chemotherapy cycles. An SAE was considered to match an AE (or vice versa) if both criteria 1 and 2 were met and any one of criteria 3-5:

1. SAE and AE both recorded for the same patient
2. SAE and AE use the same medical term
3. Date of onset (SAE) and date of assessment (AE) match exactly, or within 30 days
4. Date of assessment lies between the date of onset and date of resolution
5. Events are confirmed to have occurred during the same chemotherapy cycle (chemotherapy cycle information was collected for both AEs and SAEs)

The date of assessment can be either 30 days before or 30 days after the date of onset in order to meet criteria 3. This is because it is possible for an event to occur, meeting the criteria for an AE, but at that point not meet the criteria for an SAE (for example hospitalisation). The event could then worsen and the patient is hospitalised, therefore meeting the criteria for an SAE. The date of onset would be the date the event met the criteria for an SAE and so would be after the date of assessment. The number and percentage of events of each toxicity term that were successfully mapped to an AE (or vice versa) will be listed.

### Impact of mapping on safety analyses

The SAE dataset was used to report the annual Development Safety Update Report (DSUR) to the regulator, but the interim safety analysis for ICON8 used only the AE dataset without the SAEs dataset [6]. That comparative analysis was performed early in the trial, after the first 150 patients recruited had completed chemotherapy. To assess the impact of including any extra events identified in the mapping process, summary statistics of the number of patients that had at least one grade 3 or worse event were compared from using just the AE dataset alone (as per previously safety analyses performed) to the combined AE and SAE dataset. The difference in grade 3 to 5 severity adverse events (with 95% confidence interval) between each experimental arm and the control arm respectively was calculated. The impact on mapping on safety analyses was performed using only the original safety dataset of these first 150 patients as the interim analysis and separately for the efficacy dataset of all 1566 patients that were ultimately recruited to the trial.

## Results

**Fig. 1:**
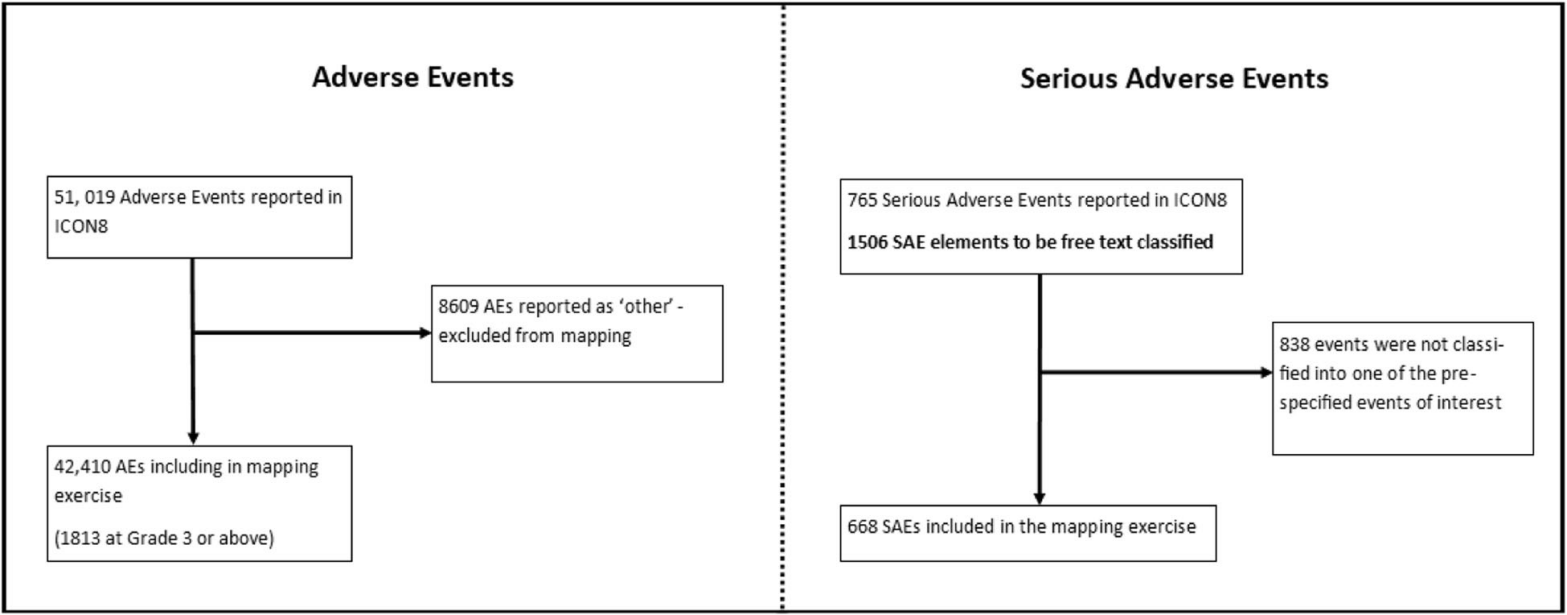
Number of adverse events and serious adverse events including in mapping exercise

The dataset for this analysis was frozen on 05 April 2018. Sites reported 51,019 adverse events for the trial’s 1566 patients. Eight thousand six hundred nine (17%) of these were reported as free-text ‘other’ AEs and were excluded in this matching analysis, leaving 42,410 AEs to which an SAE could potentially be matched. One thousand eight hundred thirteen (4%) of these AEs were at severity grade 3 or worse. A list of all AEs by body system is in Supplementary Table 1. Sites reported 765 SAEs, comprising 1506 SAE elements (main and associated). Six hundred sixty-eight (44%) of these free-text terms were classified into one of the pre-specified events of interest (Fig. 1).

Sixty-one percent (408/668) of SAE elements were matched to an already-reported AE. Vomiting was the most commonly mapped SAE to an existing AE with 75/143 (52%) matches. Most event terms classifications had at least half of the SAE elements successfully mapped to an already-reported AE (Table 2).

**Table 2:** ICON8 serious adverse events mapped to already-reported adverse events

Event classification	Total SAEs	Not matched to AE	Matched to AE
Vomiting	143	68 (48%)	75 (52%)
Diarrhoea	77	35 (46%)	42 (55%)
Nausea	68	25 (37%)	43 (63%)
Febrile neutropenia	65	31 (48%)	34 (52%)
Thromboembolic event	52	24 (46%)	28 (54%)
Fatigue	41	8 (20%)	33 (81%)
Constipation	41	8 (20%)	33 (81%)
Neutrophil count decreased	33	10 (30%)	23 (70%)
Anaemia	32	4 (13%)	28 (88%)
Pain	29	13 (45%)	16 (55%)
Rash	15	7 (47%)	8 (53%)
Dehydration	15	10 (67%)	5 (33%)
Allergic reaction	14	5 (36%)	9 (64%)
Anorexia	11	2 (18%)	9 (82%)
Platelet count decreased	10	2 (20%)	8 (80%)
Peripheral sensory neuropathy	5	1 (20%)	4 (80%)
ALT or AST elevation	4	2 (50%)	2 (50%)
Creatinine increased	2	0 (0%)	2 (100%)
Myalgia	2	1 (50%)	1 (50%)
Weight loss	2	0 (0%)	2 (100%)
White blood cell decreased	2	0 (0%)	2 (100%)
Myocardial infarction	1	1 (100%)	0 (0%)
Peripheral motor neuropathy	1	1 (100%)	0 (0%)
Hypokalaemia	1	1 (100%)	0 (0%)
Arrhythmia	1	1 (100%)	0 (0%)
Muscle weakness	1	0 (0%)	1 (100%)
**Total**	**668**	**260 (39%)**	**408 (61%)**

Supplementary Table 2 shows the level of matching of SAEs to already-reported AEs by participating centre. Of the 93 sites with at least one SAE reported, 18 did not match any SAE elements to already-reported AEs, whilst 42 sites matched at least 50% of the SAEs to an already-reported AE.

Supplementing the 51,019 AEs dataset with the SAEs elements for the same period that could not be matched with an already-reported AEs increased the safety dataset by 1086 (2%) additional events (Table 3). The proportion of patients with at least one grade 3 or worse safety events increased in all arms from 42 to 47% in the control arm and 61 to 65% and 52 to 59% in the research arms. The difference in proportions of grade 3 or worse event in the research arms compared to the control arm changed by 18% (95% confidence interval [CI] 12 to 24%) and 12% (95% CI 6 to 18%), respectively.

**Table 3:** ICON8 updated safety analysis—total events

	Standard	Research 1	Research 2	Total
**Carboplatin**	3-weekly	3-weekly	Weekly	
**Paclitaxel**	3-weekly	Weekly	Weekly	
**AE dataset only**				
Number of events	15,767	18,690	16,592	51,019
Proportion of patients with a grade 3+ event	42%	61%	52%	
Difference in grade 3+ events (95% CI)	n/a	19% (13%, 25%)	9% (3%, 15%)	
**Combined dataset**				
Additional SAE elements	300	343	443	1086
Number of events	16,067	19,033	17,005	52,105
Proportion of patients with a grade 3+ event	47%	65%	59%	
Difference in grade 3+ events (95% CI)	n/a	18% (12 to 24%)	12% (6 to 18%)	

Focusing on the first 150 patients in the initial safety analysis, 76 (2%) additional events were identified that had not previously been reported as an AE (Table 4). The proportion of patients with at least one grade 3 or worse safety event increased considerably in all research arms from 32 to 42% in the control arm and 56 to 60% and 46 to 56% in the research arms. The difference in proportions of grade 3 or worse events in the research arms compared to the control arm changed by 18% (95% CI − 1 to 37%) and 14% (95% CI − 5 to 33%) respectively.

**Table 4:** ICON8 interim safety analysis—total events

	Standard	Research 1	Research 2	Total
**Carboplatin**	3-weekly	3-weekly	Weekly	
**Paclitaxel**	3-weekly	Weekly	Weekly	
**AE dataset only**				
Number of events	1419	1705	1459	4583
Proportion of patients with a grade 3+ event	32%	56%	46%	
	–			
Difference (95% CI)	n/a	24% (5%, 43%)	14% (−5%, 33%)	
**Combined dataset**				
Additional SAE elements	20	28	28	76
Number of adverse events	**1439**	**1733**	**1487**	**4659**
Proportion of patients	42%	60%	56%	
Difference in grade 3+ events (95% CI)	n/a	18% (−1%, 37%)	14% (−5%, 33%)	

Of the 42,410 AEs included in this mapping exercise, only 535 (1%) of these could be matched to an already-reported SAE. Only one event classification, febrile neutropenia, had more than 50% of AEs matched to an already-reported SAE, at 38/75 occurrences. Nine AEs had no occurrences matched to an SAE (Supplementary Table 2, left). Focusing on those 1813 AEs with severity grade 3 or worse which were more likely to also be serious, a slightly higher proportion (200, 11%) matched to an SAE (Supplementary Table 2, right). Two event types had more than 50% of events successfully matched to an SAE; vomiting at 67% (30/45) and febrile neutropenia at 51% (38/75). Nine toxicities had none of its occurrences matched to an SAE. Supplementary Table 3 shows the level of matching of CTCAE grade 3 or worse events AEs matched to already-reported SAEs. Forty-two of 107 sites reporting at least one severe AE could not match any severe AEs to an already-reported SAE; 3/107 sites matched more than 50% of severe AEs to an already-reported SAE.

## Discussion

We found low agreement in mapping SAEs to already-reported AEs, with nearly 40% of eligible SAEs not mapped to an already-reported adverse event. Although the absolute increase in number of adverse events by combining the datasets was modest, the proportion of patients with at least one severe adverse event increased considerably. Therefore, any analyses of safety data that use only AE datasets or do not clearly account for SAE data will likely be missing important safety information.

Importantly, trials that simply add AE and SAE data for safety analyses without accounting for the element of duplicate reporting of individual adverse events may have over-reporting (double counting) of safety events in the safety analyses. Whilst the dangers of underreporting the full safety profile of a drug have been well documented [7, 8], there is undoubtedly also issues with over-reporting safety events. In an extreme case, this could lead to an efficacious drug not being offered to patients due to over-estimated safety concerns. It is accepted that where safety data is summarised as a binary analysis (for example, has a patient experienced at least one grade 3 or above event during the course of the trial, as in the ICON8 analysis), duplication of events may not be as detrimental to the analysis and its interpretation. However, when analysing safety data at a finer grained level, such as the frequency of particular safety events or their duration, it will be vital to ensure that there has not been any duplication in said events. Investigators should always be explicit about which datasets have contributed to safety analyses and, wherever possible, ensure all safety events are included whilst avoiding duplication.

We found low levels of matching routinely collected AEs to already-reported SAEs, at only 1.3%. However, this is unsurprising, because many events will not have met the criteria for seriousness. Even if one assumes that a higher proportion of severe adverse events would meet the seriousness criteria, information about seriousness was not collected routinely for AEs so assessment of missing SAEs through this approach is not straightforward. There is an understandable pressure to limit the amount of data collected from sites in order to reduce the burden, particularly in academic-led trials [9]; however, if each adverse event severity grading assessment was supplemented by ‘Did this event meet the seriousness criteria?’, adverse events and SAEs pooling would be simplified. Such a question would also serve as a reminder to sites to report events deemed to be an SAE in an expedited fashion as per the regulations.

The interpretation of the main ICON8 safety analyses with the revised datasets was unchanged. However, the interim safety analysis, performed on the first 150 patients, saw a considerable increase in severe events and a particular impact on the difference in proportions of patients with severe safety events between the first research arm and the control arms. This difference in the rate of events reported in the control arm could suggest sites do not report safety events in the control arm as readily as in research arms since the safety of this treatment is already known and understood. Such under-reporting would cause bias in assessing the comparative safety profile of treatments. This impact was less apparent in the safety analysis at the end of the trial, so this consideration may be more important for interim safety analysis and Data Monitoring Committees where these may be potential for a trial to be closed on safety grounds without good merit.

It is good practice that a reconciliation of safety data is performed. Doing this ensures that trial teams report one clear summary of safety data and therefore alleviate some of the issues discussed in this paper. However, there is often no detail given in the results of trial safety analyses to show that this has taken place. Reconciling safety sources may normally be performed towards the end of the trial, or when a DSUR is submitted (if applicable), which may not necessarily coincide with the timing of any interim analyses.

SAEs reported for the ICON8 trial were recorded as free-text events based on the CIOMs form. This free-text needed to be coded according to the AEs that were pre-specified for routine-collection. This required a large amount of free-text searching which, for this methodology project, was done by a statistician. Therefore, a potential limitation is that some events could have been missed from the classification process, particularly in the case of misspellings or unusual alternative terms. We accounted for obvious spelling errors and alternative spellings and terminology and manually checked SAEs that were not automatically classified in order to reduce this issue, but the potential for missed events remains. A potential area of future work is to consider an automated coding system based in AI-facilitated natural language processing (NLP) in order to classify free-text events according to the MedDra system (which can then be mapped across to CTCAE if so desired).

Our analysis included safety events that were one of the pre-specified AEs recorded on the toxicity section of the chemotherapy CRF. However, there was also an option for ‘other’ AEs, where a site could record any other AEs that they deemed reportable. These were recorded as free-text and excluded from this mapping exercise. Potentially some of these events could have been successfully mapped to SAEs; however, most had a low severity grade, and so may be less likely to also meet the seriousness criteria.

This was a trial of already-licenced treatments with well-known toxicity profiles in a moderately common cancer. The implications of not correctly pooling routinely collected adverse event data with as-required SAE data may be different in other settings, such as other disease types, treatments with greater or lower toxicity risks and trials with toxicity assessed during long-term follow-up and trials with more or fewer event categories routinely assessed. We have identified two further trials in prostate and gastric cancer in which we will explore this as part of a PhD project.

## Conclusions

In conclusion, AEs and SAEs are both collected in clinical trials. In cancer trials, it is often plausible that some AEs will also be recorded as an SAE, and vice versa. However, this is not always well completed and creates problems when analysing safety data and making conclusions from said data. Reporting standards should make clear which datasets were used for analyses. It may further be advisable to trials to collect additional information on AEs, to determine if these have also been reported as SAEs. This would ensure regulatory reporting requirements are being met and enable accurate pooling of both AE and SAE data for safety analyses.

## Supplementary information

**Additional file 1: Supplementary Table 1.** Free Text Categorisations. **Supplementary Table 2.** ICON8 Adverse Events Mapped to Serious Adverse Events. **Supplementary Table 3.** ICON8 Consistency Between Centres.

## Data Availability

Data will be shared according the Medical Research Council Clinical Trials Unit controlled access approach, based on the following principles: a) No data should be released that would compromise an ongoing trial or study b) There must be a strong scientific or other legitimate rationale for the data to be used for the requested purpose c) Investigators who have invested time and effort into developing a trial or study should have a period of exclusivity in which to pursue their aims with the data, before key trial data are made available to other researchers d) The resources required to process requests should not be underestimated, particularly successful requests that lead to preparing date for release, thus adequate resources must be available to comply in a timely manner or at all, and the scientific aims of the study must justify the use of such resources e) Data exchange complies with Information Governance and Data Security Policies in all the relevant countries Researchers wishing to access data from this study should contact the corresponding author in the first instance.
