# Supplementary material for: Overlap between adverse events (AEs) and serious adverse events (SAEs): a case study of a phase III cancer clinical trial
Source: Trials. 2020 Sep 17;21:802. doi: 10.1186/s13063-020-04718-z (PMC7495966; doi:10.1186/s13063-020-04718-z)
Supplement: Supplementary file 1 — Additional file 1: Supplementary Table 1. Free Text Categorisations. Supplementary Table 2. ICON8 Adverse Events Mapped to Serious Adverse Events. Supplementary Table 3. ICON8 Consistency Between Centres. [file 13063_2020_4718_MOESM1_ESM.docx]

# Comparison of Safety Data in Phase III Cancer Clinical Trials – Supplementary Appendix

Supplementary Table 1 Free Text Categorisations

| **Body System (v4)** | **CTCAE term** | **Free text searches** | **ICON8** | **STAMPEDE** | **ST03** | **Notes** |
| --- | --- | --- | --- | --- | --- | --- |
| Blood and lymphatic system disorders | Anaemia | "anaemia" "anemia" | X | X |  | CTCAE v3 uses the term 'Hemoglobin' (however STAMPEDE uses the term anaemia) |
|  | Febrile neutropenia/Infection with neutropenia | "febrile" + "neutropenia" "neutropenic" + "sepsis" "infection" + "neutropenia" "infection" + "neutrophils" "infection" + "anc" "infection" + "neutrphils" "infection" + "neutropenic" "neutropaenic" + "sepsis" | X | X | X | Event called 'febrile neutropenia' in ICON8 & STAMPEDE, 'infection with neutropenia' in ST03 |
| Cardiac disorders | Acute coronary syndrome | "acute" + "coronary" + "syndrome" | X |  |  |  |
|  | Myocardial infarction | "myocardial" + "infarction" "myocardial" + "infarct" "cardicac" + "ischemia" "cardiac" + "infarction" | X | X | X | CTCAE v3 uses the term 'cardiac ischemia/infarction' (despite both ST03 & STAMPEDE using the term myocardial infarction) |
|  | Arrhythmia* | "arrythmia" "atrial" + "fibrillation" "dysrhythmia" "fast" + "af" "atrial" + "fibrilation" "arterial" + "fibrillation" "atrial" + "flutter" "tachycardia" "arrhythmia" | X | X | X | This term was used in v3 of CTCAE, but not v4 (which is what ICON8 used). |
|  | Cardiac failure | "cardiac" + "failure" "cardiac" + "arrest" "cardiac" + "arest" "cardiopulmonary" + "arrest" "cardio" + "pulmonary" + "arrest" |  |  | X | Not in CTCAE v3 or v4 |
|  | Chest pain (angina) | "chest" + "pain" "cardiac" + "pain" "angina" |  |  | X | Not in CTCAE v3 - 'angina' is graded as 'cardiac ischemia/infarction' |
|  | Reduction in LVEF | "reduction" + "lvef" "left" + "ventricular" + "systolic" |  |  | X | CTCAE v3 uses the term 'left ventricular systolic dysfunction' |
| Ear and labyrinth disorders | Tinnitus | "tinnitus" |  |  | X |  |
| Eye disorder | Blurred vision | "blurred" + "vision" "fuzzy" + "vision" |  | X |  |  |
|  | Conjuctivitis* | "conjunctivitis" "ocular" + "surface" + "disease" |  | X |  | In CTCAE v3 (which STAMPEDE used) this is termed as 'ocular surface disease', however in v4 it is called 'conjuctivitis' |
| Gastrointestinal disorders | Constipation | "constipation" | X | X |  |  |
|  | Diarrhoea | "diarrhoea" "diarrhea" "diarrheoa" "loose" + "stools" | X | X | X |  |
|  | Dry mouth | "dry" + "mouth | X |  |  |  |
|  | Mucositis oral | "mucositis" + "oral" | X |  |  |  |
|  | Nausea | "nausea" | X | X | X |  |
|  | Vomiting | "vomiting" "vomitting" "vomtting" | X | X | X |  |
|  | Stomatitis | "stomatitis" "mucositis" |  | X | X |  |
|  | Gastrointestinal perforation | "gastrointestinal" + "perforation" "gastric" + "perforation" "gastro" + "intestinal" + "perforation" "gi" + "perforation" |  |  | X |  |
|  | Fistulae | "fistulae" "fistula" |  |  | X |  |
|  | Haemorrhage | "haemorrhage" "hemorrage" "haemorrage" "hemorrhage" haemmorhage" haemorrage" |  | X | X |  |
|  | Abdominal pain | "abdominal" + "pain" |  | X |  | Event uses the term 'pain - select' in CTCAE v3 |
|  | Dyspepsia | "heartburn" "dyspepsia" |  | X |  |  |
|  | Flatulence | "flatulence" |  | X |  |  |
| General disorders and administration site concerns | Pain/generalised pain | "pain" | X | X |  | Event uses the term 'pain - select' in CTCAE v3, 'pain' in CTCE v4 |
|  | Fatigue/Lethargy/Asthenia | "lethargy" "fatigue" "lethergic" "asthenia" "malaise" "tired" | X | X | X | Event uses the term 'fatigue' in ICON8, 'lethargy' in ST03 In STAMPEDE 'asthenia' and 'lethargy' are recorded as two separate events, however CTCAE v3 classes these as the same events. For purposes of comparison analysis, these are combined in STAMPEDE (as per CTCAE) |
|  | Fever | "fever" "pyrexia" |  | X |  |  |
|  | Flu-like symptoms | "flu" + "like" |  | X |  | Event uses the term 'flu-like syndrome' in CTCAE v3, 'flu-like symptoms' in CTCAE v4 |
|  | Oral candidiasis | "oral" + "candidiasis" |  | X |  | Not in CTCAE v3 |
|  | Fluid retention | "fluid" + "retention" "edema" |  | X |  | Not in CTCAE v3 - 'edema' is recorded |
| Hepatobiliary Disorders | Abnormal hepatic function | "abnormal" + "hepatic" "abnormal" + "liver" "abnormal" + "lft" |  | X |  | Not in CTCAE v3 |
| Immune system disorders | Allergic reaction | "allergic" "hypersensitivity" "reaction" + "paclitaxel"* "infusion" + "reaction"* | X |  | X | * Only used in ICON8 as paclitaxel reaction specific to trial, and infusion reaction included as separate event in ST03 |
|  | Acute infusion reaction | "infusion" + "reaction" "cytokine" + "release" + "syndrome" |  |  | X |  |
|  | Hypersensitivity | "hypersens" |  | X |  |  |
| Infections and infestations | Ototoxicity | "ototoxicity" "otitis" |  |  | X | CTCAE v3 separates this into external and middle ear |
|  | Infection (normal neutropenia count) | "infection" + NOT "neutropenia" "infection" + NOT "neutrophils" "infection" + NOT "anc" "infection" + NOT "neutrphis" |  |  | X | Event termed as 'infection with normal ANC or grade 1 or 2 neutrophils' in CTCAE v3 |
|  | Urinary tract infection | "urinary" + "tract" + "infection" "uti" |  | X |  |  |
|  | Rhinitis | "rhinitis" |  | X |  | Event termed as 'allergic rhinitis' in CTCAE v3 |
|  | Sinusitis | "sinusitis" |  | X |  | Not In CTCAE v3 |
|  | Upper respiratory tract infection | "upper" + "respiratory" + infection" |  | X |  | Event termed as 'infection - select' in CTCAE v3 |
| Investigations | Neutrophil count decreased/neutrophils/neutropenia | "neutrophil" + "count" + "decrease" "neutrophils" "neutropen" + NOT "febrile" "neutropen" + NOT "sepsis" "neutropenia" + NOT "infection" | X | X |  | Event uses the term 'neutrophil count decreased' in ICON8 (as per v4 CTCAE), 'neutrophils' in STAMPEDE (as per v3 CTCAE) |
|  | AST or ALT elevation | "alt" + "elevation" "alt" + "increased" "alanine" + "aminotransferase" "ast" + "elevation" "ast" + "increased" "asparate" + "aminotranferase" "alt" + "raised" | X | X |  | In STAMPEDE these are separated into two different events - Increased AST, and increased ALT (as per CTCAE) |
|  | Creatinine increased | "creatinine" + "increase" "creatinine" + "rise" "creatinine" + "raised" | X |  |  |  |
|  | Platelet count decreased | "platelet" + "count" + "decrease" "thrombocytopenia" | X | X | X |  |
|  | Weight loss | "weight" + "loss" | X |  |  |  |
|  | White blood cell decreased | "white" + "blood" + "decrease" | X |  |  |  |
|  | Liver toxicity | "liver" "kidney" |  |  | X | Not in CTCAE v3 |
|  | Bilirubin increase | "hyperbilirubin" "bilirubin" + "increase" |  | X |  | Event termed as 'bilirubin (hyperbilirubinemia) in CTCAE v3 |
| Metabolism and nutrition disorders | Anorexia | "anorexia" "loss" + "appetite" "decreased" + "appetite" | X | X | X |  |
|  | Dehydration | "dehydrat" | X |  |  |  |
|  | Hypokalaemia | "hypokalaemia" "hypokalemia" | X | X |  |  |
|  | Hypocalcaemia | "hypocalc" "reduced" + "calcium" |  | X |  |  |
|  | Hypophosphatemia | "hypophosphatemia" "reduced" + "phosphate" |  | X |  |  |
|  | Diabetes | "diabetes" "hyperglycemia" |  | X |  | Event uses the term 'hyperglycemia' in CTCAE v3, but STAMPEDE used the term diabetes |
| Musculoskeletal and connective tissue disorders | Arthralgia | "arthralgia" "joint" + "pain" | X | X |  | Event termed as 'pain - select in CTCAE v3, and 'arthralgia' in CTCAE v4 |
|  | Muscle weakness | "muscle" + "weakness" "weakness" + "both" + "legs" | X |  |  | Event termed as 'generalised muscle weakness' in CTCAE v4 |
|  | Myalgia | "myalgia" "muscle" + "pain" | X | X |  | Event termed as 'pain - muscle' in CTCAE v3, 'myalgia' in CTCAE v4 |
|  | Bone pain | "bone" + "pain" |  | X |  |  |
| Nervous system disorders | Peripheral motor neuropathy | "peripheral" + "motor" + "neuropathy" "motor" + "neuropathy" | X |  |  | ST03 uses the term 'peripheral neuropathy' and does not distinguish between sensory and motor neuropathy |
|  | Peripheral sensory neuropathy | "peripheral" + "sensory" + "neuropathy" "peripheral" + "neuropathy" + NOT "motor" "periperal" + "sensory" + "neuropathy" "sensory" + "neuropathy" | X |  | X | ST03 uses the term 'peripheral neuropathy' and does not distinguish between sensory and motor neuropathy |
|  | Loss of taste | "loss" + "taste" "dysgeusia" "taste" + "alteration" |  |  | X | Event termed as 'taste alteration (dysgeusia) in CTCAE v3 |
|  | Dizziness | "dizziness" "dizzy" |  | X |  |  |
|  | Headache | "headache" |  | X |  | Event termed as 'pain - select' in CTCAE v3 |
|  | Neurotoxicity | "neurotoxicity" |  |  | X | Not in CTCAE v3 - whole body system |
|  | Cognitive disturbance | "cognitive" + "disturb" |  | X |  |  |
| Psychiatric disorders | Insomnia | "insomnia" |  | X |  |  |
| Renal and Urinary disorders | Proteinuria | "proteinuria" |  |  | X |  |
|  | Urinary frequency | "urinary" + "frequency" "urinary" + "urgency" |  | X |  |  |
|  | Acute renal failure | "renal" + "failure" "kidney" + "failure" |  | X |  | Event uses the term 'renal failure' in CTCAE v3 |
|  | Haematuria | "hematuria" "haematuria" |  | X |  | In CTCAE v3 this is defined as 'Haemorrhage, GU', however in v4 is it defined as 'haematuria' |
|  | Renal impairment | "renal" + "impair" |  | X |  | Not in CTCAE v3 |
|  | Renal toxicity | "renal" "kidney" |  |  | X | Not in CTCAE v3 - whole body system |
| Reproductive system and breast disorders | Impotence | "impotence" |  | X |  | Not in CTCAE v3 or v4 |
|  | Breast pain | "breast" + "pain" |  | X |  |  |
|  | Breast enlargement | "breast" + "enlarge" "gynecomast" |  | X |  | Event uses thes term 'gynecomastia' in CTCAE v3, but STAMPEDE uses the term breast enlargement |
| Respiratory, thoracic and mediastinal disorders | Cough | "cough" |  | X |  |  |
|  | Dyspnoea | "dyspnea" "dyspnoea" "breathlessness" "shortness" + "breath" "sobe" |  | X |  |  |
|  | Pharyngitis | "pharyngitis" "sore" + "throat" |  | X |  | Not in CTCAE v3 |
| Skin and subcutaneous tissue disorders | Rash | "rash" | X | X |  |  |
|  | Alopecia | "alopecia" "hair" + "loss" | X |  | X |  |
|  | Nail changes | "nail" + "change" "nail" + "ridg" |  | X |  |  |
|  | PPE | "ppe" "palmar" + "plantar" "hand" + "foot" + "syndrome" |  |  | X | Event uses the term 'Rash: hand-foot skin reaction' in CTCAE v3 |
| Vascular disorders | Thromboembolic event | "thromboembolic" + "event" "pulmonary" + "emboli" "deep" + "vein" + "thrombosis" "dvt" | X |  |  |  |
|  | Pulmonary embolism* | "pulmonary" + "embolism" "pe" "p.e" "pulmonary" + "emboli" "embolism" "pulmonary" + "embolus" "pulmonary" + "emobolism" "pulmonary" + "embolis" |  |  | X | Event uses the term 'thrombosis/embolism (vascular access-related)' or 'thrombosis/thrombus/embolism' in CTCAE v3 |
|  | Hypertension | "hypertension" |  | X | X |  |
|  | Hypotension | "hypotension" |  | X |  |  |
|  | Hot flashes | "hot" + "flash" "hot" + "flush" |  | X |  |  |
|  | Transient ischaemic attack | "transient" + "ischaemic" + "attack" "cerebrovascular" + "ischemia" |  |  | X | Event termed as 'CNS cerebrovascular ischemia' in CTCAE v3 |
|  | Cerebrovascular accident | "cerbrovascular" + "accident" "stroke" |  |  | X | Event termed as 'CNS cerebrovascular ischemia' in CTCAE v3 |
|  | Other arterial thromboembolic event | "arterial" + "thromboembolic" |  |  | X | Not in CTCAE v3 |
|  | Deep vein thrombosis | "deep" + "vein" + "thrombosis" "dvt" |  |  | X | Event uses the term 'thrombosis/embolism (vascular access-related)' or 'thrombosis/thrombus/embolism' in CTCAE v3 |
|  | Other venous thromboembolic event | "venous" + thromboembolic" "venous" + thrombosis" |  |  | X |  |

Supplementary Table 2 - ICON8 Adverse Events Mapped to Serious Adverse Events

|  | **All Adverse Events** | | | **Grade 3+ Adverse Events** | | |
| --- | --- | --- | --- | --- | --- | --- |
| **Event name** | **Total** | **Not matched to SAE** | **Matched to SAE** | **Total** | **Not matched to SAE** | **Matched to SAE** |
| Alopecia | 6237 | 6237 (100.0%) | 0 (0.0%) | 0 | n/a | n/a |
| Fatigue | 5428 | 5369 (98.9%) | 59 (1.1%) | 65 | 57 (87.7%) | 8 (12.3%) |
| Anaemia | 4258 | 4208 (98.8%) | 50 (1.2%) | 157 | 145 (92.4%) | 12 (7.6%) |
| Peripheral sensory neuropathy | 3643 | 3637 (99.8%) | 6 (0.2%) | 61 | 57 (93.4%) | 4 (6.6%) |
| Neutrophil count decreased | 2755 | 2719 (98.7%) | 36 (1.3%) | 678 | 652 (96.2%) | 26 (3.8%) |
| Constipation | 2662 | 2621 (98.5%) | 41 (1.5%) | 17 | 14 (82.4%) | 3 (17.6%) |
| White blood cell decreased | 2556 | 2554 (99.9%) | 2 (0.1%) | 251 | 250 (99.6%) | 1 (0.4%) |
| Nausea | 2299 | 2244 (97.6%) | 55 (2.4%) | 36 | 27 (75.0%) | 9 (25.0%) |
| Pain | 1799 | 1783 (99.1%) | 16 (0.9%) | 44 | 41 (93.2%) | 3 (6.8%) |
| Platelet count decreased | 1225 | 1213 (99.0%) | 12 (1.0%) | 117 | 111 (94.9%) | 6 (5.1%) |
| Diarrhoea | 1189 | 1133 (95.3%) | 56 (4.7%) | 40 | 24 (60.0%) | 16 (40.0%) |
| ALT or AST elevation | 964 | 962 (99.8%) | 2 (0.2%) | 17 | 16 (94.1%) | 1 (5.9%) |
| Mucositis oral | 952 | 952 (100.0%) | 0 (0.0%) | 2 | 2 (100.0%) | 0 (0.0%) |
| Rash | 836 | 825 (98.7%) | 11 (1.3%) | 11 | 10 (90.9%) | 1 (9.1%) |
| Arthralgia | 801 | 801 (100.0%) | 0 (0.0%) | 9 | 9 (100.0%) | 0 (0.0%) |
| Anorexia | 750 | 741 (98.8%) | 9 (1.2%) | 14 | 10 (71.4%) | 4 (28.6%) |
| Vomiting | 704 | 616 (87.5%) | 88 (12.5%) | 45 | 15 (33.3%) | 30 (66.7%) |
| Myalgia | 620 | 619 (99.8%) | 1 (0.2%) | 7 | 6 (85.7%) | 1 (14.3%) |
| Dry mouth | 554 | 554 (100.0%) | 0 (0.0%) | 0 | n/a | n/a |
| Peripheral motor neuropathy | 364 | 364 (100.0%) | 0 (0.0%) | 5 | 5 (100.0%) | 0 (0.0%) |
| Weight loss | 356 | 353 (99.2%) | 3 (0.8%) | 2 | 2 (100.0%) | 0 (0.0%) |
| Muscle weakness | 306 | 303 (99.0%) | 3 (1.0%) | 5 | 5 (100.0%) | 0 (0.0%) |
| Creatinine increased | 295 | 292 (99.0%) | 3 (1.0%) | 5 | 5 (100.0%) | 0 (0.0%) |
| Allergic reaction | 259 | 249 (96.1%) | 10 (3.9%) | 21 | 14 (66.7%) | 7 (33.3%) |
| Hypokalaemia | 219 | 219 (100.0%) | 0 (0.0%) | 23 | 23 (100.0%) | 0 (0.0%) |
| Thromboembolic event | 180 | 152 (84.4%) | 28 (15.6%) | 87 | 62 (71.3%) | 25 (28.7%) |
| Dehydration | 80 | 74 (92.5%) | 6 (7.5%) | 17 | 12 (70.6%) | 5 (29.4%) |
| Febrile neutropenia | 75 | 37 (49.3%) | 38 (50.7%) | 75 | 37 (49.3%) | 38 (50.7%) |
| Arrhythmia | 42 | 42 (100.0%) | 0 (0.0%) | 1 | 1 (100.0%) | 0 (0.0%) |
| Myocardial infarction | 1 | 1 (100.0%) | 0 (0.0%) | 1 | 1 (100.0%) | 0 (0.0%) |
| Acute coronary syndrome | 1 | 1 (100.0%) | 0 (0.0%) | 0 | n/a | n/a |
| **Total** | **42410** | **41875 (98.7%)** | **535 (1.3%)** | **1813** | **1613 (89.0%)** | **200 (11.0%)** |

Supplementary Table 3 - ICON8 Consistency Between Centres

| **Centre** | **SAEs successfully matched to AEs** | **AEs successfully matched to SAEs (Grade 3+ events only)** |
| --- | --- | --- |
| Centre_27 | 1 (100.0%) | 1 (33.3%) |
| Centre_36 | 2 (100.0%) | 0 (0.0%) |
| Centre_53 | 1 (100.0%) | 1 (25.0%) |
| Centre_54 | 2 (100.0%) | 1 (10.0%) |
| Centre_58 | 2 (100.0%) | 0 (0.0%) |
| Centre_104 | 1 (100.0%) | 2 (100.0%) |
| Centre_120 | 1 (100.0%) | 1 (20.0%) |
| Centre_334 | 1 (100.0%) | No AEs reported |
| Centre_521 | 2 (100.0%) | No AEs reported |
| Centre_723 | 1 (100.0%) | 0 (0.0%) |
| Centre_729 | 6 (100.0%) | 6 (20.7%) |
| Centre_867 | 3 (100.0%) | 4 (36.4%) |
| Centre_302 | 18 (78.3%) | 5 (12.5%) |
| Centre_35 | 17 (77.3%) | 12 (11.5%) |
| Centre_832 | 15 (75.0%) | 8 (27.6%) |
| Centre_22 | 5 (71.4%) | 4 (13.3%) |
| Centre_117 | 5 (71.4%) | 1 (11.1%) |
| Centre_45 | 8 (66.7%) | 1 (6.3%) |
| Centre_96 | 6 (66.7%) | 2 (33.3%) |
| Centre_102 | 2 (66.7%) | 0 (0.0%) |
| Centre_462 | 6 (66.7%) | 1 (6.3%) |
| Centre_812 | 2 (66.7%) | 1 (12.5%) |
| Centre_23 | 3 (60.0%) | 3 (18.8%) |
| Centre_439 | 6 (60.0%) | 3 (9.1%) |
| Centre_454 | 6 (60.0%) | 1 (3.1%) |
| Centre_555 | 18 (60.0%) | 11 (21.6%) |
| Centre_3 | 13 (59.1%) | 4 (11.4%) |
| Centre_268 | 13 (59.1%) | 6 (13.0%) |
| Centre_26 | 7 (58.3%) | 4 (8.7%) |
| Centre_179 | 8 (57.1%) | 4 (40.0%) |
| Centre_548 | 4 (57.1%) | 4 (66.7%) |
| Centre_44 | 9 (56.3%) | 3 (37.5%) |
| Centre_520 | 5 (55.6%) | 3 (33.3%) |
| Centre_78 | 12 (54.5%) | 9 (12.9%) |
| Centre_422 | 7 (53.8%) | 0 (0.0%) |
| Centre_108 | 12 (52.2%) | 8 (9.4%) |
| Centre_125 | 2 (50.0%) | 1 (33.3%) |
| Centre_450 | 1 (50.0%) | 1 (7.1%) |
| Centre_453 | 1 (50.0%) | 0 (0.0%) |
| Centre_716 | 1 (50.0%) | 1 (4.3%) |
| Centre_896 | 2 (50.0%) | 1 (16.7%) |
| Centre_973 | 1 (50.0%) | 1 (1.7%) |
| Centre_32 | 20 (47.6%) | 7 (12.1%) |
| Centre_321 | 6 (46.2%) | 2 (22.2%) |
| Centre_48 | 5 (45.5%) | 1 (16.7%) |
| Centre_74 | 7 (43.8%) | 4 (14.8%) |
| Centre_41 | 10 (43.5%) | 8 (20.5%) |
| Centre_81 | 3 (42.9%) | 3 (33.3%) |
| Centre_92 | 3 (42.9%) | 2 (66.7%) |
| Centre_131 | 3 (42.9%) | 2 (22.2%) |
| Centre_715 | 6 (42.9%) | 5 (22.7%) |
| Centre_47 | 10 (41.7%) | 3 (27.3%) |
| Centre_768 | 2 (40.0%) | 0 (0.0%) |
| Centre_52 | 5 (38.5%) | 3 (25.0%) |
| Centre_24 | 6 (35.3%) | 0 (0.0%) |
| Centre_43 | 10 (34.5%) | 3 (8.3%) |
| Centre_20 | 8 (33.3%) | 2 (12.5%) |
| Centre_51 | 1 (33.3%) | 1 (11.1%) |
| Centre_87 | 4 (33.3%) | 3 (27.3%) |
| Centre_113 | 2 (33.3%) | 2 (7.7%) |
| Centre_151 | 2 (33.3%) | 1 (3.6%) |
| Centre_243 | 13 (33.3%) | 6 (16.2%) |
| Centre_711 | 13 (33.3%) | 10 (10.8%) |
| Centre_75 | 7 (30.4%) | 3 (10.7%) |
| Centre_830 | 5 (27.8%) | 3 (7.5%) |
| Centre_55 | 3 (27.3%) | 1 (9.1%) |
| Centre_17 | 4 (26.7%) | 0 (0.0%) |
| Centre_5 | 2 (25.0%) | 2 (20.0%) |
| Centre_66 | 4 (22.2%) | 2 (14.3%) |
| Centre_18 | 1 (20.0%) | 0 (0.0%) |
| Centre_286 | 1 (20.0%) | 0 (0.0%) |
| Centre_19 | 1 (14.3%) | 0 (0.0%) |
| Centre_271 | 1 (14.3%) | 1 (16.7%) |
| Centre_575 | 1 (12.5%) | 1 (33.3%) |
| Centre_132 | 1 (10.0%) | 0 (0.0%) |
| Centre_8 | 0 (0.0%) | 0 (0.0%) |
| Centre_11 | 0 (0.0%) | 0 (0.0%) |
| Centre_30 | 0 (0.0%) | 0 (0.0%) |
| Centre_34 | 0 (0.0%) | 0 (0.0%) |
| Centre_38 | 0 (0.0%) | 0 (0.0%) |
| Centre_62 | 0 (0.0%) | 0 (0.0%) |
| Centre_99 | 0 (0.0%) | 0 (0.0%) |
| Centre_119 | 0 (0.0%) | 0 (0.0%) |
| Centre_168 | 0 (0.0%) | 0 (0.0%) |
| Centre_171 | 0 (0.0%) | 0 (0.0%) |
| Centre_237 | 0 (0.0%) | 0 (0.0%) |
| Centre_382 | 0 (0.0%) | 0 (0.0%) |
| Centre_403 | 0 (0.0%) | 0 (0.0%) |
| Centre_512 | 0 (0.0%) | 0 (0.0%) |
| Centre_771 | 0 (0.0%) | 0 (0.0%) |
| Centre_804 | 0 (0.0%) | 0 (0.0%) |
| Centre_907 | 0 (0.0%) | 0 (0.0%) |
| Centre_925 | 0 (0.0%) | 0 (0.0%) |
| Centre_2 | No SAEs reported | 0 (0.0%) |
| Centre_14 | No SAEs reported | 0 (0.0%) |
| Centre_28 | No SAEs reported | 0 (0.0%) |
| Centre_31 | No SAEs reported | 0 (0.0%) |
| Centre_40 | No SAEs reported | 0 (0.0%) |
| Centre_113 | No SAEs reported | 0 (0.0%) |
| Centre_114 | No SAEs reported | 0 (0.0%) |
| Centre_116 | No SAEs reported | 0 (0.0%) |
| Centre_311 | No SAEs reported | 0 (0.0%) |
| Centre_372 | No SAEs reported | 0 (0.0%) |
| Centre_636 | No SAEs reported | 0 (0.0%) |
| Centre_727 | No SAEs reported | 0 (0.0%) |
| Centre_797 | No SAEs reported | 0 (0.0%) |
| Centre_800 | No SAEs reported | 0 (0.0%) |
| Centre_831 | No SAEs reported | 0 (0.0%) |
| Centre_994 | No SAEs reported | 0 (0.0%) |
